# Visual Performance after a Unilateral or Bilateral Implantation of Enlarged Depth-of-Focus Intraocular Lens in Patients with Cataract: A Prospective Clinical Trial

**DOI:** 10.1155/2019/2163809

**Published:** 2019-03-04

**Authors:** Se Hyun Choi, Hyo kyung Lee, Chang Ho Yoon, Mee Kum Kim

**Affiliations:** ^1^Department of Ophthalmology, Seoul National University College of Medicine, Seoul, Republic of Korea; ^2^Laboratory of Ocular Regenerative Medicine and Immunology, Seoul Artificial Eye Center, Seoul National University Hospital Biomedical Research Institute, Seoul, Republic of Korea

## Abstract

**Purpose:**

To investigate visual performances after a unilateral or bilateral implantation of enlarged depth-of-focus intraocular lens in patients with cataract.

**Methods:**

In this prospective study, uneventful phacoemulsification and TECNIS® Symfony intraocular lens implantation were performed in 20 eyes of 17 patients. At postoperative 1, 4, and 12 weeks, the logarithm of the minimal angle of resolution visual acuity at far, intermediate, and near distances and the spherical equivalent in manifest refraction and automated refraction were measured. A questionnaire was used to investigate glare, spectacle dependency, and satisfaction at 12 weeks. The mean numerical error and mean absolute error were compared between intraocular lens formulas to assess the best-fit formula.

**Results:**

The logarithm of the minimal angle of resolution visual acuity significantly improved to 0.02 at far, 0.02 at intermediate, and 0.27 at near distances at 12 weeks (*p* < 0.05). Spherical equivalent was −0.79 D on automated refraction and was significantly lower than −0.26 D measured on manifest refraction. Patients' satisfaction score was 9.06, 8.94, and 6.65 for far, intermediate, and near distances, respectively. Near glasses were required in 5 patients and 2 patients complained of photic phenomenon. Visual performances were not significantly different between bilateral and unilateral implanted patients. No patients reported bilateral imbalance due to unilateral surgery. The mean numerical error was closest to 0 D using the Barrett Universal II formula. The mean absolute error was not significantly different between these formulas.

**Conclusion:**

Unilateral or bilateral implantation of the enlarged depth-of-focus intraocular lens seems to be equally effective in improving visual performances in patients with cataract.

## 1. Introduction

With the advancement of the intraocular lens (IOL), cataract surgery has become an operation not only to replace an opaque crystalline lens with a clear intraocular lens but also a procedure to correct the refractive error and presbyopia [1, 2]. Initially, bifocal IOLs were available, but as the demand for intermediate vision inpatients using computers increased, trifocal IOLs became popular [3, 4]. However, bifocal or trifocal IOLs have limitation that the working distances between these fixed focal points are involved in suboptimal visual acuity [5–7]. A newly developed enlarged depth-of-focus (EDOF) IOL may provide better optical quality on the whole addition range than fixed multifocal IOLs with a proprietary achromatic diffractive surface designed to correct chromatic aberration and a echelette feature to extend the range of vision [5, 8].

Recently, a few reports comparing TECNIS® Symfony IOL with other multifocal IOLs have been published and reported good intermediate visual outcomes compared to other IOLs [9, 10]. However, most previous studies included only patients who underwent bilateral surgery and did not analyse the refractive changes [11, 12]. In addition, few reports investigated the subjective satisfaction with unilateral bifocal IOL implantation but not with EDOF lens [13, 14]. This study aimed to report visual performances including refractive outcomes, subjective satisfaction, and spectacle independence in patients undergoing unilateral or bilateral TECNIS® Symfony IOL implantation.

## 2. Materials and Methods

### 2.1. Patients

This prospective study adhered to the tenets of the Declaration of Helsinki and was approved by the Institutional Review Board of Seoul National University Hospital (IRB no. 1612-133-820). The study was conducted from February 2017 to May 2018. Twenty eyes of 17 patients who visited ophthalmology outpatient clinic of Seoul National University Hospital for the cataract surgery and were willing to participate in the study were included. All included participants signed a consent form.

Inclusion criteria were as follows: (1) patients with cataracts confirmed by slit-lamp examination preoperatively; (2) patients who underwent uneventful unilateral or bilateral cataract surgery with implantation of the EDOF IOL (TECNIS® Symfony or TECNIS® Symfony Toric); (3) age of ≥16 years; (4) patients with regular corneal astigmatism of ≤0.75 Diopter (D) for TECNIS® Symfony IOL and between 1.0 and 3.62 D for TECNIS® Symfony toric IOL.

Exclusion criteria were as follows: (1) pregnant or nursing women; (2) presence of other ocular disease that may affect the stability of the lens capsule such as pseudoexfoliation syndrome, traumatic cataract, and Marfan syndrome; (3) presence of other ocular diseases that are expected to have a poor final visual acuity of <20/30 after the cataract surgery; (4) pupil abnormality; (5) white cataract; (6) systemic or ocular medication that may affect the visual acuity; (7) previous refractive surgery; and (8) patients participating in other clinical trials during the study.

### 2.2. Surgical Technique

All cataract surgeries were performed by a single surgeon (M.K.K.). After the conventional phacoemulsification through the temporal clear corneal incision, TECNIS® Symfony or TECNIS® Symfony Toric IOLs were implanted in the capsular bag. Postoperatively, topical fluorometholone acetate ophthalmic suspension (Flarex®, Alcon, Alcon Laboratories Inc., Fort Worth, TX, USA) were administered four times a day for 1 week, topical levofloxacin 0.5% (Cravit®, Santen Pharmaceutical, Osaka, Japan) eye drops were administered four times a day for 1 month, and topical bromfenac sodium ophthalmic solution 0.1% (Bronuck®, Taejoon Pharm, Seoul, Korea) was instilled twice a day for 2 months.

### 2.3. Clinical Evaluation

The primary outcomes of the study were improvement of visual acuity at near, intermediate, and far. Secondary outcomes included presence of photic phenomena and spectacle independence for daily tasks.

Corrected distance visual acuities (CDVA) were measured before the surgery, and 1, 4, and 12 weeks after the surgery to evaluate the safety index. Uncorrected visual acuities (UCVA) were measured at far (5 m), intermediate (66 cm), and near (40 cm) distances preoperatively, and then 1, 4, and 12 weeks postoperatively. Snellen visual acuities were converted to the logarithm of minimal angle of resolution (LogMAR) for statistical analysis. Thorough ophthalmic examinations including intraocular pressure, slit-lamp examination, and keratometry were conducted at every follow-up.

Automated refraction (AR) and manifest refraction (MR) were performed at postoperative 1, 4, and 12 weeks. The IOL master 700 (Carl Zeiss Meditec AG, Jena, Germany) was used to calculate SRK/T, Holladay 2, Haigis, and Hoffer Q formulas. The Barrett Universal II and Hill-RBF formulas were calculated by inputting the values measured by IOL master 700 measurements into the online software. The refraction prediction error was calculated as the difference between the actual postoperative manifest refraction and the predicted refraction for each formula. The mean numerical error (MNE) was defined as the arithmetic mean of the prediction errors, and the mean absolute error (MAE) was defined as the mean of the magnitude of the prediction errors.

At postoperative 4 and 12 weeks, the contrast sensitivity test (VCTS 6500, Vistech Consultants Inc., Dayton, OH, USA) and ALC glare test (v1.3, ALC Clinic, Seoul, Korea) were performed as previously reported [15, 16].

To evaluate the patients' subjective outcome, a questionnaire was used as previously described [11]. Patient's satisfaction score in near, intermediate, and far tasks was measured from 0 (unsatisfactory) to 10 (very satisfactory) at postoperative 4 and 12 weeks. Patients were asked regarding their spectacle dependencies at near, far, and intermediate distances with 0%, 25%, 50%, 75%, and 100% of time. Photic phenomena were evaluated using an open question whether the patients had any problems with their vision such as glare or halos.

In order to increase compliance of the patients, each outpatient visit was informed in advance. All data were recorded in electronic medical charts, and all data were collected by two independent ophthalmologists.

### 2.4. Statistical Analysis

All statistical analyses were conducted using Prism software (GraphPad Prism version 7, Inc. La Jolla, CA, USA). To compare quantitative variable between the data at two time points or between unilateral and bilateral implantation, a nonparametric Mann–Whitney *U* test was used, and to compare the changes over time within a group, the paired *t*-test was used. To compare two time points, Wilcoxon matched-pairs signed-rank test was used, and to compare three or more time points, repeated measures analysis of variance (rANOVA) was used. Data were presented as the mean ± standard deviation (SD), and the differences were considered significant at *p* < 0.05.

## 3. Results

A total of 20 eyes of 17 patients were enrolled, and all patients completed a 12-week follow-up. Among them, 3 patients (*n* = 6 eyes) underwent bilateral EDOF lens implantation and the other 14 patients underwent unilateral EDOF lens implantation. The demographic and preoperative data of patients are summarised in Table 1. Of the 14 patients who underwent unilateral surgery, the opposite eye was excluded because it did not meet the inclusion criteria. Two eyes were pseudophakic, 8 eyes were without significant cataract, 2 eyes had epiretinal membrane, 1 eye had severe astigmatism, and 1 eye had history of previous refractive surgery. The spherical equivalent (SE) of the opposite eyes was −0.26 ± 2.22 D.

### 3.1. Visual Performances

**Figure 1:**
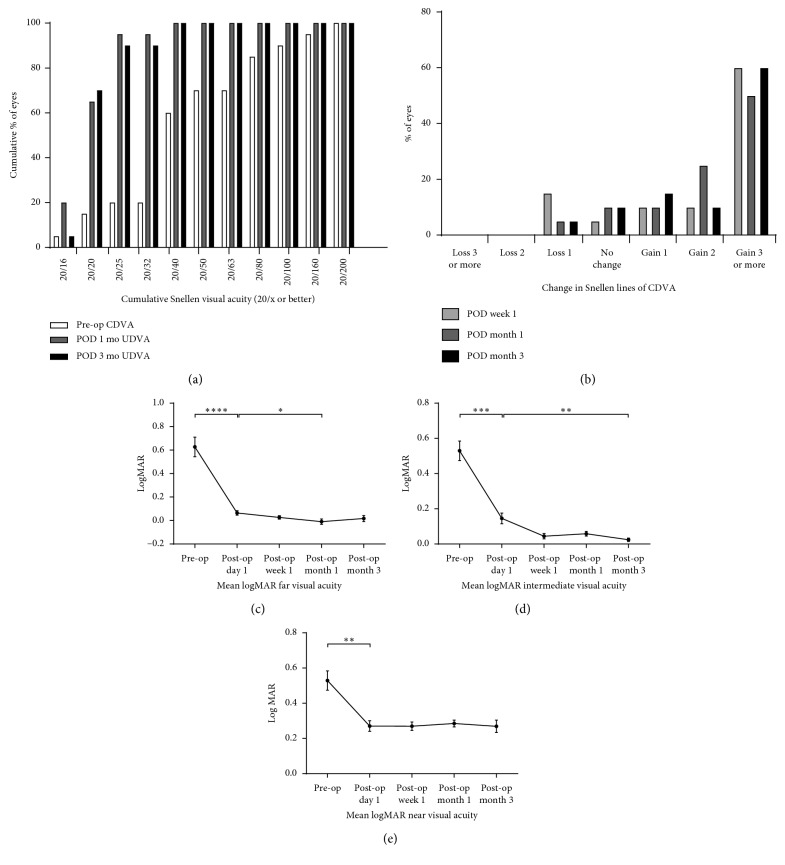
Visual outcome after the implantation of TECNIS® Symfony IOL. (a) Histogram showing preoperative best-corrected distance visual acuity and postoperative uncorrected distance visual acuity. (b) Histogram showing change in snellen lines of corrected distance visual acuity. (c–e) Graphs showing the change in LogMAR visual acuity at far, intermediate, and near distances at postoperative 1 day and 1, 4, and 12 weeks.

**Figure 2:**
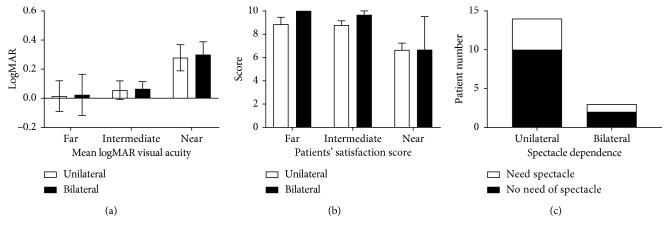
Comparison of binocular and monocular surgery. (a) Mean LogMAR visual acuity of unilateral and bilateral surgery group at distance, intermediate, and near at 12 weeks. (b) Mean patients' satisfaction score of unilateral and bilateral surgery group. (c) Histogram comparing the number of patients in the unilateral and bilateral surgery group who needed spectacles at near distance.

Eighteen eyes (90%) showed uncorrected distance visual acuity (UDVA) of 20/25 or better at 12 weeks postoperatively, and 14 eyes (70%) showed UDVA of 20/20 or better at postoperative 12 weeks (Figure 1(a)). Fifteen eyes (75%) gained two or more lines of CDVA after the surgery, and only one eye (5%) lost one line postoperatively (Figure 1(b)). LogMAR UCVA was improved at far (*p* < 0.0001, rANOVA), intermediate (*p*=0.0003, rANOVA), and near (*p*=0.0063, rANOVA) distances at postoperative day 1 compared with the values preoperatively and remained stable until 3 months after the surgery (Figures 1(c)–1(e)). No difference in LogMAR UCVA was observed between bilateral and unilateral group sat far (*p*=0.5859, Mann–Whitney *U* test), intermediate (*p*=0.6424, Mann–Whitney *U* test), and near (*p*=0.7045, Mann–Whitney *U* test) distances at postoperative 12 weeks (Figure 2(a)).

### 3.2. Refractive Errors

**Figure 3:**
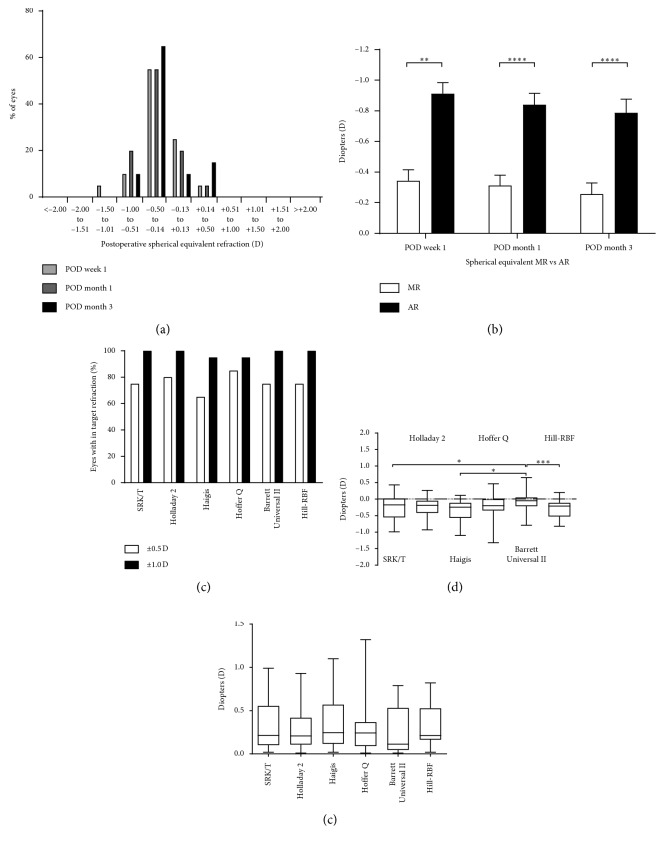
Refractive outcome after the implantation of TECNIS® Symfony IOL. (a) Histogram showing postoperative spherical equivalent (SE) refraction measured by manifest refraction at 1, 4, and 12 weeks postoperatively. (b) Histogram showing the differences in SE between manifest refraction and automated refraction. (c) Histogram showing the percentage of eyes within ± 0.5 D and ± 1.0 D for the target refraction. (d) Histogram showing mean numerical error using SRK/T, Holladay 2, Haigis, Hoffer Q, Barrett Universal II, and Hill-RBF formulas. (e) Histogram showing mean absolute error using SRK/T, Holladay 2, Haigis, Hoffer Q, Barrett Universal II, and Hill-RBF formulas.

Preoperative spherical equivalent measured by MR was −1.74 ± 3.14 D, and the postoperative spherical equivalent was −0.34 ± 0.32 D, −0.31 ± 0.30 D, and −0.26 ± 0.33 at postoperative 1, 4, and 12 weeks, respectively. Eighteen eyes (90%) showed SE within ± 0.5 D, and all eyes showed SE within ± 1.0 D at 12 weeks after the surgery (Figure 3(a)). Postoperative SE measured by AR shifted significantly toward myopic compared with SE measured by MR at postoperative 1 (*p*=0.0041, paired *t*-test), 4 (*p*=0.0001, paired *t*-test), and 12 weeks (*p*=0.0001, paired *t*-test) (Figure 3(b)). The mean difference was −0.53 ± 0.30 D for SE at postoperative 12 weeks.

In three eyes with TECNIS® Symfony Toric IOL implantation, preoperative corneal astigmatism was measured as 2.30 ± 0.95 D with IOL master, 1.90 ± 0.91 D with topography, and 1.97 ± 0.84 D with keratometry and decreased to residual refractive cylinder of 0.33 ± 0.29 D at postoperative 12 weeks.

All formulas met the benchmark criteria [17] for postoperative refraction results (Figure 3(c)). The Barrett Universal II formula had a highest (closest to 0 D)MNE of −0.08 ± 0.35 D, which was significantly higher than Hill-RBF (0.0002, rANOVA), SRK/T (0.0175, rANOVA) and Haigis (0.0190, rANOVA) formulas (Figure 3(d)). The Barrett Universal II formula also had the lowest MAE of 0.25 ± 0.25 D but was not significantly different from other formulas (Figure 3(e)).

### 3.3. Contrast Sensitivity Test and Glare Test

**Figure 4:**
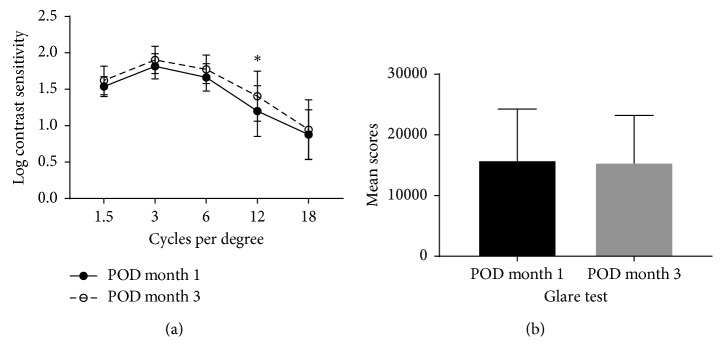
Contrast sensitivity test and glare test. (a) Mean contrast sensitivity (in logarithmic scale) at postoperative 1 and 3 months. (b) Mean scores of the glare test at postoperative 1 and 3 months.

Contrast sensitivity values at all spatial frequencies were within normal limits at 4 and 12 weeks postoperatively. Contrast sensitivity increased to12 cycles per degree at postoperative 12 weeks compared with that of the postoperative 4 weeks (*p*=0.0273, paired *t*-test) (Figure 4(a)). Mean scores of the glare test was 15796.9 ± 8459.5 at postoperative 4 weeks and 15418.4 ± 7788.3 at postoperative 12 weeks. The difference between the two time points was not statistically significant (*p*=0.7197, Wilcoxon matched-pairs signed-rank test) (Figure 4(b)).

### 3.4. Patient Satisfaction, Spectacle Dependence, and Photic Phenomena

Patients' satisfaction score was 9.06 ± 2.08 for far, 8.94 ± 1.30 for intermediate, and 6.65 ± 2.67 for near-distance vision at postoperative 12 weeks. Patients' satisfaction scores were significantly higher at far (*p*=0.0327, rANOVA) and intermediate (*p*=0.0031, rANOVA) distance vision than at near vision. No significant difference in patients' satisfaction score was observed between bilateral and unilateral surgery groups at all distances (Figure 2(b)).

All patients did not need a spectacle at far and intermediate distances at 12 weeks postoperatively. In near tasks, two of the unilateral surgery patients required wearing spectacles for <50% of the time, and two unilateral surgery patients and one bilateral surgery patient required spectacles for >50% of the time. No clinically significant difference in spectacle dependency was observed between unilateral and bilateral surgery groups (>0.9999, Fisher's exact test) (Figure 2(c)).

Only two patients complained of glare at postoperative 12 weeks. Only one patient reported bilateral imbalance due to unilateral surgery (7.2%).

## 4. Discussion

In the eyes implanted with TECNIS® Symfony IOL, the UCVA at far, intermediate, and near distances was significantly improved just a day after the surgery and well maintained for 3 months. As with the previous results, [18, 19] which ranged from 0.08 to 0.00, excellent mean logMAR UDVA of 0.02 ± 0.11 was also obtained at 12 weeks in our study. This confirms the ability of the EDOF IOL to gain near vision without sacrificing distance vision. The mean logMAR uncorrected intermediate visual acuity (UIVA) of 0.02 and uncorrected near visual acuity (UNVA) of 0.27 were also within the normal range of visual acuities as previously reported [19, 20]. Along with previous studies in which the logMAR UNVA ranged from 0.30 to 0.20, [21] the monocular UNVA in our study, when compared with monocular UNVA in other multifocal IOLs measured at 40 cm, was inferior to that of +3.0 D bifocal IOL, AcrySof® IQ ReSTOR® (Alcon Laboratories, Inc., Fort Worth, TX, USA), ranging from 0.15 to 0.05, [3, 10] but compatible with UNVA of 0.23 with AT Lisa® tri 839MP (Carl Zeiss Meditec AG, Jena, Germany) or 0.24 with Fine Vision IOL (Physiol, Liege, Belgium) [22, 23]. The TECNIS® Symfony IOL is considered to be better suited for patients who spend more time on intermediate-distance tasks, such as computer work, rather than near-distance tasks, such as reading or using mobile phones. In patients with a demand for near-field work that is closer than 40 cm, a micro-monovision technique can be adopted as reported by Cochener et al. [11].

Regarding the refractive outcome, patients achieved SE within ± 0.5 D in 18 eyes (90%) and within ± 1.0 D in all eyes. To the authors' best knowledge, no report has been conducted on the distribution of SE; therefore, our results could not be compared with previous studies. Instead, the mean SE value of all previous reports was within ± 0.5 D [10, 11, 19]. This excellent refraction predictability is believed to have contributed to good distance visual acuity. One of the important findings revealed in this study is that the SE measured by AR was significantly more myopic than the SE measured by MR, with a mean difference of −0.53 D. Munoz et al. reported that AR showed poor correlation for spherical values with a trend toward more negative values in refractive multifocal IOL [24]. Bissen-Miyajima et al. reported a good correlation between AR and MR in a full aperture diffractive TECNIS® bifocal IOL with pupil independency [25]. However, TECNIS® Symfony IOL in a pupil-dependent posterior diffractive design has a dominant peak at the intermediate point, and a pupil of <2 mm produces continuous peak from far to intermediate focus, which may result in a difference between AR and MR [26, 27].

To achieve optimal performance of multifocal IOL, accurate IOL power calculation is essential. To the authors' knowledge, no previous report has investigated a formula that fit best in TECNIS® Symfony IOL, and Cochener et al. and Pedrotti et al. only used the SRK/T formula [10, 11, 18]. Reitblat et al. reported high accuracy of 86.3 to 93.2% within ± 0.5 D and 100% within ±1.0 D without any significant difference between all formulas after implanting AcrySof® IQ ReSTOR® IOL (Alcon Laboratories, Inc., Fort Worth, TX, USA) [28]. SRK/T, Holladay 2, Haigis, Hoffer Q, Barrett Universal II, and Hill-RBF formulas used in this study also showed high accuracy of 65 to 85% within ± 0.5 D and 95 to 100% within ±1.0 D. Based on our results, the Barrett Universal II formula showed an MNE that is closest to 0 D and the tendency towards lowest MAE; therefore, the Barrett Universal II formula could be considered as a first reference.

Most previous studies using multifocal IOL have been performed bilaterally, and reports on unilateral surgery are limited [7, 12]. According to previous reports, unilateral multifocal IOL implantation could achieve high spectacle independence and stereoacuity, but significantly less than that after the bilateral surgery [29, 30]. Unlike the previous studies, the patient satisfaction score was as high as 9.06 for far, 8.94 for intermediate, and 6.65 for near vision, even though the ratio of unilateral surgery patients was 82.4%, showing no significant difference in patient satisfaction score or spectacle dependence between binocular and monocular surgery patients. In addition, 92.8% of patients did not complain of binocular disparity in patients who underwent the unilateral surgery. Therefore, EDOF IOL can be effectively used in patients who have presbyopia and unilateral cataract and require good intermediate vision for computer or navigator use.

## 5. Conclusions

In conclusion, TECNIS® Symfony IOL restores excellent visual acuity especially at far and intermediate distances. High accuracy can be achieved by using third- and fourth-generation IOL formulas and evaluating postoperative refractions by MR. The satisfaction levels were high in both unilateral and bilateral patients with low spectacle dependence and no binocular disparity in unilateral surgery patients. Therefore, TECNIS® Symfony IOL can be effectively used in not only bilateral cataract patients but also in unilateral patients with presbyopia.

## Figures and Tables

**Table 1:** Demographics and preoperative visual acuity and refractive data.

Parameters	Values

Age (years)	
Mean ± SD	55.05 ± 13.38
Range	17–67
Sex (male : female)	7 : 10
Laterality (unilateral : bilateral)	14 : 3
Preoperative visual acuity (LogMAR, mean ± SD)	
Distance (corrected)	0.34 ± 0.29
Distance (uncorrected)	0.63 ± 0.37
Intermediate	0.53 ± 0.25
Near	0.53 ± 0.25
Preoperative spherical equivalent (D, mean ± SD)	−1.74 ± 3.14

SD: standard deviation; LogMAR: logarithm of the minimal angle of resolution; D: diopters.

## Data Availability

The data used to support the findings of this study are available from the corresponding author upon request.
